# Social determinants of health and exclusive breastfeeding: a longitudinal study

**DOI:** 10.17533/udea.iee.v42n3e16

**Published:** 2024-11-02

**Authors:** Ananda Larisse Bezerra da Silva, Elizabete Regina Araújo Oliveira, Wanessa Lacerda Poton, Andreia Soprani dos Santos, Susana Bubach, Maria Helena Monteiro de Barros Miotto

**Affiliations:** 1 PhD. Nurse. Email: anandalarisse@hotmail.com. Corresponding Author. https://orcid.org/0000-0003-0061-1103 Universidade Federal do Espírito Santo Brazil anandalarisse@hotmail.com; 2 PhD. Associate Professor. Email: elizabete_regina@hotmail.com. https://orcid.org/0000-0002-6616-4273 Universidade Federal do Espírito Santo Brazil elizabete_regina@hotmail.com; 3 PhD. . Email: wanessa.poton@uvv.br. https://orcid.org/0000-0001-5849-0653 Universidade de Vila Velha Brazil wanessa.poton@uvv.br; 4 PhD. Full Professor. Email: andreia.s.santos@ufes.br. https://orcid.org/0000-0002-4377-6517 Universidade Federal do Espírito Santo Brazil andreia.s.santos@ufes.br; 5 PhD. Full Professor. Email: susana.bubach@ufes.br. https://orcid.org/0000-0002-7190-5275 Universidade Federal do Espírito Santo Brazil susana.bubach@ufes.br; 6 PhD. Full Professor. Email: mhmiotto@terra.com.br. https://orcid.org/0000-0002-3227-7608 Universidade Federal do Espírito Santo Brazil mhmiotto@terra.com.br; 7 Federal University of Espirito Santo, Vitoria, Espirito Santo, Brazil. Universidade Federal do Espírito Santo Federal University of Espirito Santo Vitoria Espirito Santo Brazil; 8 Federal University of Amapá, Macapá, Amapá, Brazil. Universidade Federal do Amapá Federal University of Amapá Macapá Amapá Brazil; 9 Vila Velha University, Vila Velha, Espirito Santo, Brazil. Universidade de Vila Velha Vila Velha University Vila Velha Espirito Santo Brazil; 10 Federal University of Espirito Santo, São Mateus, Espirito Santo, Brazil. Universidade Federal do Espírito Santo Federal University of Espirito Santo São Mateus Espirito Santo Brazil

**Keywords:** breastfeeding, social determinants of health, newborn., lactancia materna, determinantes sociales de la salud, recién nacido., aleitamento materno, determinantes sociais de saúde, recém-nascido.

## Abstract

**Objective.:**

To analyze the social determinants of health of exclusive breastfeeding on the 7^th^ and 27^th^ day after delivery.

**Methods.:**

A longitudinal, multicenter study with a quantitative approach was conducted with postpartum women and newborns in three maternity hospitals in the state of Espírito Santo (Brazil). The sample consisted of 2,325 and 1,819 mother/baby pairs on the 7^th^ and 27^th^ day, respectively. The variables that most influence women in exclusive breastfeeding were assessed by logistic regression.

**Results.:**

The rate of exclusive breastfeeding seven days after delivery was 80.7% and 79.2% on the 27^th^ day. The social determinants of health up to the 27^th^ day after delivery that were related to exclusive breastfeeding aimed at the mother were: mother's age up to 19 years (OR 1.9); and in newborns, the following stood out: full-term baby (OR 2.2) and no difficulty in EBF (OR=8.8).

**Conclusion.:**

The relationship between social determinants of health such as maternal age, gestational age of the baby at birth and difficulties in breastfeeding influence the practice of EBF.

## Resumen

**Objetivo.:**

Analizar los determinantes sociales de la lactancia materna exclusiva (LME) en los días 7 y 27 después del parto.

**Métodos.:**

Estudio longitudinal, multicéntrico, con abordaje cuantitativo, realizado con puérperas y recién nacidos en tres maternidades del estado de Espírito Santo (Brasil). La muestra consistió en 2325 y 1819 binomios madre/bebé en el 7º y 27º día, respectivamente. Las variables que más influyeron en las mujeres en la LME fueron evaluadas por regresión logística**.**

**Resultados.:**

La tasa de LME a los siete días del parto fue del 80.7% y del 79.2% al 27º día. El determinante social de la salud hasta el 27º día tras el parto que se relacionó con la LME para la madre fue la edad hasta 19 años (OR 1.9); y para el recién nacido: el haber nacido a término (OR 2.2) y no tener ninguna dificultad para amamantar (OR=8.8).

**Conclusión.:**

La relación entre determinantes sociales de la salud como la edad materna, la edad gestacional del bebé al nacer y las dificultades para la lactancia materna influyen en la práctica de la LME.

## Resumo

**Objetivo.:**

Analisar os determinantes sociais de saúde do aleitamento materno exclusivo (AME) no 7º e no 27º dia após o parto.

**Métodos.:**

Estudo longitudinal, multicêntrico, com abordagem quantitativa realizado com puérperas e recém-nascidos, em três maternidades do estado do Espírito Santo (Brasil). A amostra foi composta por 2325 e 1819 binômios mãe/bebê no 7º e no 27º dia, respectivamente. As variáveis que mais influenciam as mulheres na amamentação exclusiva foram avaliadas por regressão logística.

**Resultados.:**

A taxa de aleitamento materno exclusivo sete dias após o parto foi de 80.7% e de 79.2% no 27º dia. Os determinantes sociais de saúde até o 27º dia após o parto que estiveram relacionados ao aleitamento materno exclusivo voltados para a mãe foram: idade da mãe até 19 anos (OR 1.9); e no recém-nascido destacaram-se: bebê nascido à termo (OR 2.2) e não ter dificuldade no AME (OR=8.8).

**Conclusão.:**

A relação dos determinantes sociais de saúde como idade materna, idade gestacional do bebê ao nascer e dificuldades na amamentação influenciam na prática do AME.

## Introduction

Exclusive breastfeeding (EBF) in the first 6 months of life is considered the most complete diet for newborns, as it provides all the energy, vitamins, and minerals that infants need. After this period, breastfeeding must continue until at least 2 years of age, even after the introduction of solid foods.^1^ This is because it is the most economical and effective form of protection against infant mortality, diarrheal diseases, respiratory infections, allergies, and other conditions, in addition to being a protective factor against maternal breast cancer and promoting better orofacial development in children.^2^^,^^3^ The National Study on Child Feeding and Nutrition (ENANI) carried out in 2019 showed that, despite Brazil's progress in breastfeeding, less than half of children aged 0 to 6 months were being exclusively breastfed (45.7%)^4^. This data contradicts the global target presented in the 2030 Agenda for Sustainable Development Goals, which proposes to reach at least 50% of EBF in the first 6 months of life by 2025 and 70% by 2030.^5^ In addition, the prevalence of continued breastfeeding in the first year of life (among children aged 12 to 23 months) in the country was 43.6%, with the median duration of EBF being three months, according to ENANI-2019.^4^

There are several factors related to the choice of not breastfeeding or stopping breastfeeding early. Studies suggest that conditions such as previous obesity or overweight, low education level, cesarean section, perception of insufficient breast milk, maternal illness, refusal to breastfeed, return to work or school, infant illness and cultural factors directly influence this decision.^6^^-^^8^ In addition to the primary role of health care provided to mothers, from prenatal care, delivery, puerperium until the first months of the baby's life, where nursing plays a leading role in care, mainly through education and health promotion in primary health care.^9^ In view of this, it is important to be aware of the social factors that determine the health of infants and mothers. Thus, theorists such as Dahlgren and Whitehead^10^ describe the social determinants of health as factors related to the population's living and working conditions that influence its health status. The model described by them is widely used, precisely because it addresses the relationships between social factors and individual and collective health.^10^ In addition to this broader understanding of the social determinants of health, in an attempt to understand more deeply the factors that affect breastfeeding, the Lancet Breastfeeding Series Group, based on a systematic review, proposed a conceptual model to identify the components of an environment conducive to breastfeeding, classifying the determinants into three categories: 1) structural; 2) environmental; and 3) individual. This framework provides a theoretical basis for exploring the specific determinants of breastfeeding in different sociocultural contexts.^3^^,^^8^ Despite the large number of studies on the SDH (Social Determinants of Health) of EBF, few address EBF adherence on the 7^th^ and 27^th^ days after birth, which justifies the question of this research: what are the social determinants of health of exclusive breastfeeding on the 7^th^ and 27^th^ days after birth? Therefore, it is essential to understand the barriers that EBF faces and to identify the nuances of this process, so that it is possible to outline actions directed to the needs of each population. Thus, this study aims to analyze the social determinants of health of exclusive breastfeeding on the 7^th^ and 27^th^ day after delivery. 

## Methods

Type of study, population, and sample. This is a longitudinal, multicenter study with a quantitative approach that used data from the Viver Project.^10^ This study was conducted with postpartum women and newborns in two maternity hospitals in the metropolitan region and in one maternity hospital in the northern region of the state of Espírito Santo, in the southeast of Brazil. The maternity hospitals that participated in the study were selected based on the following aspects: a) those that performed the most deliveries in the region; b) being located in one of the two health regions; c) having a diversity of care, with 80% to 100% coverage by the Unified Health System and 100% private or covered by supplementary health insurance. The population of the Viver Project corresponds to all mothers and children born alive or who died in the perinatal period. They were invited to participate in the study from August 2019 to January 2020, resulting in an initial population of 5,369 mother/baby binomials. The study sample was a census sample, as it included the entire population of mothers of children born in the three maternity hospitals that met the selection criteria, covering the entire universe investigated during the study period.

Techniques and Procedures. To conduct this study, the sample investigated was composed of two moments: 7^th^ and 27^th^ days after delivery, including two samples of mother/baby dyads, with 2,325 and 1,819, respectively. With a percentage of losses and refusals of 21.7% (*n* = 506) between the two periods. To capture the study sample, an interview was initially conducted in the maternity hospital, before hospital discharge. From that moment in question, for this study, sociodemographic and prenatal-related variables were used. The independent variables investigated in the study were: maternal characteristics, at both time points, consisting of age group, marital status, race, maternal years of schooling, maternal employment, family income, socioeconomic status according to the Brazilian Association of Research Companies, which uses the purchasing power of families as a classification criterion, from highest (A) to lowest (E), number of people living in the same household, use of alcohol and/or tobacco and use of illicit drugs. Variables related to breastfeeding: feeding and deleterious oral habits investigated on the 7^th^ and 27^th^ days of the babies' lives. Data collection was performed by telephone survey, using a previously prepared research instrument, on the 7^th^ and 27^th^ days of the child's life. However, in order to minimize segment losses, a characteristic of this type of study, the first monitoring was carried out between the 7^th^ and 10^th^ day after birth and the second between the 27^th^ and 30^th^ day, by a team trained for this purpose.

Data analysis. A descriptive analysis of the data was carried out using frequency tables with numbers and percentages. The variables that most influence women in exclusive breastfeeding were assessed by logistic regression. The significance level adopted was 5%. The statistical package IBM SPSS 20 - was used for this analysis.

Ethical aspects. The study was approved by the Human Research Ethics Committee of the University of Vila Velha (CAAE 02503018.0.0000.5064). The Informed Consent Form was signed by the mother or guardian of the child, while still in the maternity ward, and they were instructed that they would be contacted by telephone to continue the research.

## Results

In total, 2,325 and 1,819 postpartum women were interviewed on the 7^th^ and 27^th^ days, respectively. The predominant age range was between 20 and 34 years, at both times, 69.5% and 68.7%. Of these, the majority lived with a partner, 82.4% and 82.7%, respectively and 48.7% declared themselves to be brown. Regarding years of study, 55.7% and 58.1% studied for 12 years or more, and 53.8% and 56.3% had a job. The predominant family income was up to two minimum wages, representing 45.8% and 44% of the times investigated. It was found that 39% and 40.2% of the study samples belonged to socioeconomic class B and lived with four to six people in the same household, corresponding to 51.9% and 51.7%. There is no statistical significance between the two moments, that is, the samples from the two periods investigated are equivalent (Table 1).

**Table 1:** Maternal data on the 7^th^ and 27^th^ days of the newborn's life

Characteristic	7^th^ day (*n*=2325)		27^th^ day (*n*=1819)		***p*-value**
	*n*	%	*n*	%
Age group					
Up to 19 years	270	11.6	204	11.2	0.571
20 - 34 years	1617	69.5	1249	68.7	
35 years or older	438	18.8	366	20.1	
Marital status					
Lives with partner	1916	82.4	1504	82.7	0.973
Does not live with partner	268	11.5	206	11.3	
Not declared	141	6.1	109	6.0	
Race					
White	648	27.9	503	27.7	0.888
Mixed	1133	48.7	886	48.7	
Black	433	18.6	342	18.8	
Yellow	16	0.7	14	0.8	
Indigenous	2	0.1	0	0.0	
Not declared	93	4.0	74	4.1	
Years of education					
Up to 4 years	19	0.8	14	0.8	0.567
5 - 8 years	224	9.6	158	8.7	
9 - 11 years	507	21.8	388	21.3	
12 years or more	1295	55.7	1057	58.1	
Not declared	280	12.0	202	11.1	
Works					
Yes	1250	53.8	1025	56.3	0.248
No	1059	45.5	783	43.0	
Not declared	16	0.7	11	0.6	
Family income in minimum wages					
Up to 1	485	20.9	357	19.6	0.853
Between 1 and 2	580	24.9	444	24.4	
Between 2 - 3	401	17.2	326	17.9	
Between 3 - 4	172	7.4	139	7.6	
Between 4 - 5	205	8.8	177	9.7	
More than 5	259	11.1	211	11.6	
Not declared	223	9.6	165	9.1	
Socioeconomic status					
A	381	16.4	317	17.4	0.156
B	907	39.0	732	40.2	
C	893	38.4	683	37.5	
D	118	5.1	72	4.0	
E	5	0.2	0	0.0	
Not declared	21	0.9	15	0.8	
People living in the household					
0 - 3	984	42.3	779	42.8	0.968
4 - 6	1206	51.9	940	51.7	
7 or more	84	3.6	63	3.5	
Not declared	51	2.2	37	2.0	
Total	2325	100.0	1819	100.0	

Of the 80.7%, 79.2% remained on EBF on the 7^th^ day and 27^th^ day on 79.2%. 19.3% on the 7^th^ day, and 20.8% received complementary feeding on the 27^th^ day. Of these, 86% on the 7^th^ day and 83.4% on the 27^th^ day were fed with human milk and industrialized formula (Table 2). Regarding difficulty in breastfeeding, 89.1% and 78.2% did not present this difficulty on the 7^th^ and 27^th^ days (Table 2). The data focused on deleterious oral habits investigated on the 7^th^ and 27^th^ days after birth showed that 79.6% and 73.8%, respectively, did not perform finger sucking. On the 7^th^ day, 66.9% did not use a pacifier, a practice that decreased to 57.4% on the 27^th^ day. The non-use of a bottle was characterized by 91% on the 7^th^ day and 76.1% on the 27^th^ day (Table 2).

**Table 2:** Data on breastfeeding, feeding and harmful oral habits of babies on the 7^th^ and 27^th^ days after delivery

Characteristic	7 days after delivery		27 days after delivery		***p*-value**
	*n*	%	*n*	%	
Exclusive breastfeeding					
Yes	1876	80.7	1441	79.2	<0.0001
No	449	19.3	378	20.8	
Not declared	1113	-	1619	-	
Complementary feeding					
Industrialized formula	32	14.0	61	16.6	<0.0001
Human milk + formula	197	86.0	307	83.4	
Human milk	0	0.0	0	0.0	
Not declared	220	-	10	-	
Difficulty breastfeeding					
Yes	505	21.8	195	10.9	<0.0001
No	1815	78.2	1593	89.1	
Not declared	1118	-	1650	-	
Digital sucking					
Yes	478	20.4	478	26.2	<0.0001
No	1860	79.6	1347	73.8	
Not declared	1100	-	1613	-	
Baby uses a pacifier					
Yes	774	33.1	775	42.6	<0.0001
No	1561	66.9	1046	57.4	
Not declared	1103	-	1617	-	
Use of bottle					
Yes	209	9.0	434	23.9	<0.0001
No	2122	91.0	1379	76.1	
Not declared	1107	-	1625	-

Regarding EBF on the 7^th^ day and the determinants related to mothers, Table 3 shows a relationship with marital status. Mothers with a partner were more likely to maintain EBF (adjusted OR = 1.86; 95% CI = 1.28-2.72) than those without a partner. In addition, the relationship between EBF and alcohol and/or tobacco consumption and not using illicit drugs was also significant. It was revealed that women who consumed alcohol and/or tobacco and who did not use illicit drugs had 1.82 and 2.99 chances of practicing EBF, respectively (adjusted OR = 1.82; 95% CI = 1.15 - 2.87; adjusted OR = 2.99; 95% CI = 1.24 - 7.17).

**Table 3:** Maternal data and the relationship between exclusive breastfeeding seven days after delivery

Characteristic	Without EBF		With EBF		Sig.	OR	Adjusted OR
	*n*	%	*n*	%			
Age group							
Up to 19 years	47	17.4	223	82.6	0.138	1.154	1.441 (0.890 - 2.335)
20 years or more	402	19.6	1653	80.4			
Marital status							
With partner	359	18.7	1557	81.3	0.001	1.563	1.869 (1.280 - 2.728)
Without partner	71	26.5	197	73.5			
Race							
White	129	19.9	519	80.1	0.783	1.078	1.043 (0.775 - 1.403)
Non-white	297	18.8	1287	81.3			
Years of education							
Up to 11 years	138	18.4	612	81.6	0.685	1.066	1.065 (0.786 - 1.443)
12 years or more	251	19.4	1044	80.6			
Works							
Yes	242	19.4	1008	80.6	0.620	1.006	1.078 (0.801 - 1.451)
No	204	19.3	855	80.7			
Family income							
Up to 2 MW	213	20.0	852	80.0	0.142	1.100	1.288 0.919 - 1.805
Over 2 MW	192	18.5	845	81.5			
Socioeconomic status							
AB	254	19.7	1034	80.3	0.983	1.047	1.004 (0.727 - 1.384)
CDE	193	19.0	823	81.0			
Use of alcohol and/or tobacco							
Yes	53	17.2	255	82.8	0.010	1.175	1.820 (1.153 - 2.874)
No	396	19.6	1621	80.4			
Use of illicit drugs							
No	411	18.7	1784	81.3	0.014	2.026	2.993 (1.249 - 7.172)
Yes	14	31.8	30	68.2

EBF = Exclusive breastfeeding; OR = Odds Ratio; MW = Minimum wage.

The logistic regression between exclusive breastfeeding on the 7^th^ day and data related to the newborn and care provided during the prenatal period showed that children born with normal weight had 2.52 (adjusted OR = 2.52; 95% CI = 1.48-4.31) chances of practicing EBF than those born with low weight, and mothers who did not have difficulties breastfeeding had 2.701 chances of maintaining EBF than those who had this difficulty (adjusted OR = 2.70; 95% CI = 2.07-3.56) (Table 4).

**Table 4:** Relationship between exclusive breastfeeding seven days after delivery and data related to the newborn and prenatal care

Characteristic	Without EBF		With EBF		Sig.	OR	Adjusted OR
	*n*	%	*n*	%			
Sex NB							
Female	220	20.1	877	79.9	0.390	1.094	1.116 (0.869 - 1.432)
Male	229	18.6	999	81.4			
Newborn weight							
Normal weight			409		18.4	1812	81.6	0.001	2.769	2.526 (1.480 - 4.313)
Low weight			40		38.5	64	61.5			
Gestational age							
Preterm			33		32.7	68	67.3	0.280	2.110	1.378 (0.770 - 2.466)
Full term			408		18.7	1775	81.3			
Primiparous							
Yes			170		21.1	635	78.9	0.517	1.252	2.030 (0.238 - 17.27)
No			211		17.6	987	82.4			
Educational activity							
Yes			100		17.8	461	82.2	0.124	1.139	1.267 (0.937 - 1.714)
No			343		19.8	1388	80.2			
Guidance on EBF							
Yes			395		19.1	1671	80.9	0.268	1.069	1.250 (0.842 - 1.856)
No			45		20.1	179	79.9			
Previous EBF experience							
Yes			176		16.7	879	83.3	0.620	1.330	1.718 (0.202 - 14.62)
No			171		21.0	642	79.0			
Difficulty in EBF							
No			258		14.3	1541	85.7	<0.001	3.086	2.701 (2.079 - 3.561)
Yes			169		34.1	327	65.9			

NB = Newborn; EBF = Exclusive breastfeeding; OR = Odds ratio.

The age group of the mothers analyzed on the 27^th^ day after delivery was shown to be a relevant determinant regarding EBF; mothers up to 19 years old were 1.934 times more likely to practice EBF than those aged 20 or older (adjusted OR = 1.93; 95% CI = 1.07-3.49) (Table 5).

**Table 5:** Relationship between exclusive breastfeeding twenty-seven days after delivery and data related to the mothers participating in the study

Characteristic	Without EBF		With EBF			Sig.		OR	Adjusted OR
N%	%	N	%
Age group				
Up to 19 years	34	16.7	170	83.3	0.029	1.353	1.934 (1.070 - 3.495)
20 years or more	344	21.3	1271	78.7			
Marital status							
With partner	313	20.8	1191	79.2	0.404	1.064	1.209 (0.775 - 1.886)
Without partner	45	21.8	161	78.2			
Race							
White	121	24.1	382	75.9	0.259	1.330	1.196 (0.876 - 1.633)
Non-white	239	19.2	1003	80.8			
Years of education							
Up to 11 years	118	21.1	442	78.9	0.459	1.002	1.133 (0.814 - 1.578)
12 years or more	223	21.1	834	78.9			
Works							
Yes	209	20.4	816	79.6	0.246	1.058	1.211 (0.876 - 1.674)
No	167	21.3	616	78.7			
Family income							
Up to 2 MW	145	18.1	656	81.9	0.625	1.305	1.097 (0.757 - 1.588)
Over 2 MW	191	22.4	662	77.6			
Socioeconomic status							
AB	237	22.6	812	77.4	0.269	1.305	1.222 (0.856 - 1.746)
CDE	138	18.3	617	81.7			
Use of alcohol and/or tobacco							
Yes	49	20.3	192	79.4	0.231	1.032	1.337 (0.831 - 2.149)
No	329	20.8	1249	79.2			
Use of illicit drugs							
Yes	8	22.2	28	77.8	0.777	1.095	1.179 (0.378 - 3.672)
No	355	20.7	1360	79.3

EBF = Exclusive breastfeeding; OR = Odds ratio; MW = Minimum wage.

Newborns born at term were 2.28 times more likely to practice EBF than those born prematurely (adjusted OR = 2.28; 95% CI = 1.18-4.40). And mothers who reported no difficulty breastfeeding were 8.827 times more likely to maintain EBF on the 27^th^ day after delivery than those who reported this difficulty (adjusted OR = 8.82; 95% CI = 5.98-13.02) (Table 6).

**Table 6:** Relationship between exclusive breastfeeding twenty-seven days after delivery and data related to the newborn and prenatal care

Characteristic	Without EBF		With EBF		Sig.	OR	Adjusted OR
N	%	N	%
Sex of newborn							
Female	178	21.2	663	78.8	0.819	1.044	1.035 (0.769 - 1.394)
Male	200	20.4	778	79.6			
Weight of newborn							
Low weight	28	31.5	61	68.5	0.545	1.810	1.224 (0.636 - 2.357)
Normal weight	350	20.2	1380	79.8			
Gestational age							
Premature	22	28.9	54	71.1	0.014	1.572	2.284 (1.184 - 4.404)
Full term	352	20.6	1359	79.4			
Primiparous							
Yes	123	19.4	510	80.6	0.669	1.062	1.670 (0.159 - 17.49)
No	189	20.4	738	79.6			
Educational activity							
Yes	93	20.9	352	79.1	0.599	1.009	1.097 (0.777 - 1.549)
No	280	20.8	1069	79.2			
Guidance on EBF							
Yes	352	21.6	1281	78.4	0.081	1.647	1.679 (0.939 - 3.003)
No	23	14.3	138	85.7			
Previous EBF experience							
Yes	145	17.9	666	82.1	0.588	1.106	1.913 (0.183 - 19.99)
No	124	19.4	515	80.6			
Difficulty with EBF							
Yes	114	58.8	80	41.2	<0.001	8.475	8.827 (5.983 - 13.02)
No	228	14.4	1359	85.6

NB = Newborn; EBF = Exclusive breastfeeding; OR = Odds ratio.

## Discussion

There are many determinants that affect exclusive breastfeeding, influencing the decision-making of nursing mothers about whether or not to continue with this practice. The EBF rate (80.7% and 79.2%) at both times analyzed, 7^th^ and 27^th^ day after delivery, was satisfactory. The different pro-breastfeeding approaches that are carried out during prenatal care may have contributed to achieving these results. A study showed that 87% of mothers had six or more prenatal consultations, and a scoping review that analyzed the health determinants of EBF reinforced that prenatal care and the number of consultations carried out during this period are determinants for the success of EBF,^12^^,^^13^ ​​in addition to the lack of difficulty in breastfeeding (78.2% and 89.1%), present in the periods investigated. This determinant is addressed both during prenatal care and, more intensively, during the postpartum period, mainly through nursing care, in which breasts are assessed and guidance is provided regarding possible changes that may occur at the beginning of breastfeeding and how to deal with them. It is known that no anatomical type of nipple can prevent breastfeeding, although its malformation may make it difficult for the baby to latch on correctly. This difficulty can be overcome through scientific and technical knowledge on the part of the health team, as well as knowledge regarding the lactation process.^14^

The marital status of the women studied was a determining factor in EBF on the 7^th^ day after delivery. Those who had a partner were more likely to maintain EBF on the 7^th^ day after delivery than those without this support network. This finding corroborates that found in other studies. In Nigeria, when investigating the importance of a support network for exclusive breastfeeding in the first months of an infant's life, it was shown that support from a partner is crucial for the continuation of this practice.^15^ This was also significant in a study conducted in China (OR = 1.91) when the father and close friends provided support in the first month after birth^8^. This finding reinforces that the presence of a husband/partner as support for the mother and newborn contributes to the practice and maintenance of exclusive breastfeeding.

The relationship between alcohol and/or tobacco use and EBF on the 7^th^ day after delivery appeared to be a determinant in this period. As well as not using illicit drugs. It is confirmed that in the second moment, that is, in the perinatal period, the determinant of alcohol and/or tobacco use was not relevant for the analysis. In this context, a study that investigated the outcome of breastfeeding in drug-using mothers on the 7^th^, 15^th^, and 30^th^ day after delivery identified a mean EBF of 28.8 days, and mothers who used legal or illegal drugs and continued to be monitored after delivery maintained EBF.^16^ The Brazilian Federation of Gynecology and Obstetrics Associations (Febrasgo) and the Ministry of Health contraindicate breastfeeding when the mother is using illegal drugs such as cocaine, heroin, and marijuana, as these and other drugs can pass through the mother's milk and harm the baby, in addition to altering the mother's cognitive behavior, posing a risk to the necessary care for their children.^17^^,^^18^ The consumption of legal and illegal substances during breastfeeding should be addressed in the context of prenatal and postpartum care, emphasizing the harm this practice causes to the mother/baby binomial. New studies are suggested that can analyze this relationship, alcohol and breastfeeding in more depth in order to explain such causality.

Low birth weight and nipple problems were negatively associated with exclusive breastfeeding in a multicenter study conducted in China.^6^ In the study in question, children born with normal weight were more likely to maintain EBF on the 7^th^ day after delivery. Therefore, normal birth weight is a positive determinant of EBF, since the opposite, low birth weight, can lead to the early introduction of infant formulas, without acceptable clinical justification. In these cases, it is extremely important to evaluate other conditions, such as correct nipple latch when the baby is breastfeeding, and clinical evaluation of the mother's breasts, among others.^19^ Studies have identified that previous successful breastfeeding and knowledge about its benefits are factors that are associated with EBF, unlike the findings of this study, which did not find a relationship between previous experience and EBF.^8^^,^^20^ However, women who did not have difficulty breastfeeding until the 7^th^ day after delivery were more likely to maintain EBF. 

Regarding EBF on the 27^th^ day after delivery, maternal age up to 19 years was a significant determinant. A recent study in India, however, showed that maternal age was negatively associated with exclusive breastfeeding.^16^ A negative association between maternal age and EBF was also found in another study.^6^ Thus, regarding maternal age, the data are in line with the literature, which highlights that adolescent mothers are more likely to wean early. However, the data found raise important points that may justify this finding, such as the role of an effective support network, from the hospital, family, community and, mainly, primary health care, which welcomes and provides specialized assistance to these adolescent mothers.^20^

In addition to these factors, gestational age was shown to be crucial for the success of breastfeeding, since children who were born at term were more likely to maintain EBF when compared to those born prematurely (OR = 1.572). This finding is supported by other studies.^21^ The determinant of not having difficulty breastfeeding was a protective factor at both times investigated. This determinant investigated nipple fissures, adequate breastfeeding technique, correct position and latch, among other problems identified by the mothers. Therefore, it is concluded that the mothers were successful in the care provided both during pregnancy and during the postpartum period. Studies have shown that counseling and guidance on breastfeeding are a strong strategy for promoting, protecting, and supporting breastfeeding.^9^ In addition to the role of nursing in this care, being the main professional category that offers support and assistance to mothers at this time.^9^

Among the limitations of the study, we highlight the number of mothers who continued to respond to the telephone survey on the 27^th^ day after delivery, even with all the strategies to reach these participants, which reduced the sample for the targeted monitoring and the lack of monitoring of the sample investigated until the sixth month of life of the babies, the period suggested for EBF. This was not possible due to problems in contacting the participants of the study, which took place during the COVID-19 pandemic.

Conclusion. The relationship between social determinants of health, such as marital status, maternal lifestyle habits, birth weight of the baby, and difficulties in breastfeeding, influences the practice of EBF. Understanding and comprehending the social determinants of health can favor greater adherence to EBF, in addition to contributing to greater knowledge and improvement of nursing practice in the context of breastfeeding.
